# Severe left ventricular dilatation and mitral regurgitation secondary to ALCAPA in childhood: a case report

**DOI:** 10.47487/apcyccv.v6i4.535

**Published:** 2025-12-29

**Authors:** Christie Villasante-Villalta, Diego Davila-Flores, Renee Montesinos-Segura, Zoila Rodriguez-Urtega, Judith Miranda-Rojas, José Cornejo-Acevedo, Fernando Vargas Peláez, Fernando Chavarri-Velarde

**Affiliations:** 1 Instituto Nacional Cardiovascular-INCOR, EsSalud, Lima, Peru. Instituto Nacional Cardiovascular-INCOR EsSalud Lima Peru; 2 Servicio de Cardiología Pediátrica, Instituto Nacional Cardiovascular-INCOR, EsSalud, Lima, Peru. Servicio de Cardiología Pediátrica Instituto Nacional Cardiovascular-INCOR EsSalud Lima Peru; 3 Servicio de Cirugía Cardiovascular Pediátrica, Instituto Nacional Cardiovascular-INCOR, EsSalud, Lima, Peru. Servicio de Cirugía Cardiovascular Pediátrica Instituto Nacional Cardiovascular-INCOR EsSalud Lima Peru

**Keywords:** Bland White Garland Syndrome, Dilated Cardiomyopathy, Mitral Valve Inssuficiency, Cardiac Surgery, Síndrome de Bland White Garland, Miocardiopatía Dilatada, Insuficiencia de la Válvula Mitral, Cirugía Cardíaca

## Abstract

Anomalous origin of the left coronary artery from the pulmonary artery (ALCAPA) is a rare congenital heart defect, with an estimated incidence of 1 in 300,000 live births and a mortality rate approaching 90% within the first year of life if left untreated. We present the case of a three-year-old boy with progressive dyspnea, paroxysmal tachycardia, and poor weight gain, initially diagnosed with severe left ventricular dilatation and severe mitral regurgitation. Transthoracic echocardiography and cardiac computed tomography angiography confirmed the diagnosis of ALCAPA. Surgical correction included left coronary artery reimplantation and mitral valve repair. The patient was discharged without complications. At a two-year follow-up, he remained asymptomatic, with preserved left ventricular function and mild mitral regurgitation. This case highlights the importance of advanced imaging in the diagnosis and the role of surgical intervention in improving outcomes in this potentially fatal condition.

## RESUMEN

El origen anómalo de la arteria coronaria izquierda desde la arteria pulmonar (ALCAPA) es una cardiopatía congénita infrecuente, con una incidencia estimada de 1 por cada 300 000 nacidos vivos y una mortalidad que alcanza el 90% en el primer año si no se corrige quirúrgicamente. Presentamos el caso de un niño de tres años con disnea progresiva, taquicardia paroxística y escasa ganancia ponderal, inicialmente diagnosticado con dilatación severa del ventrículo izquierdo e insuficiencia mitral severa. La ecocardiografía transtorácica y la angiotomografía cardíaca confirmaron el diagnóstico de ALCAPA. Se realizó cirugía de reimplante de la arteria coronaria izquierda y plastia mitral, siendo dado de alta sin complicaciones. En el seguimiento a dos años, el paciente permanece asintomático, con función ventricular preservada e insuficiencia mitral leve. Este caso subraya la relevancia del diagnóstico por imágenes y del tratamiento quirúrgico en esta entidad potencialmente letal.

## Introduction

The anomalous origin of the left coronary artery from the pulmonary artery (ALCAPA) is a rare congenital malformation, with an estimated prevalence of 1 per 300,000 live births, accounting for approximately 0.5% of all congenital heart diseases ^1^^,^^2^. Its pathophysiology is characterised by retrograde flow from the left coronary artery into the pulmonary artery, leading to chronic myocardial ischaemia, left ventricular dysfunction, and secondary mitral regurgitation ^1^^,^^3^. Early surgical correction improves survival, promotes recovery of ventricular function, and reduces the severity of mitral regurgitation ^4^. In the absence of surgery, mortality may reach up to 90% within the first year of life ^1^.

We present the case of a three-year-old patient with progressive dyspnoea and growth retardation, initially diagnosed with left ventricular dilatation and severe mitral regurgitation of unclear aetiology. Imaging studies confirmed the diagnosis of ALCAPA, and the patient underwent coronary reimplantation and mitral valve repair, with a favourable clinical course. This case highlights the importance of considering ALCAPA in paediatric patients presenting with left ventricular dilatation and mitral regurgitation of uncertain origin, and underscores the value of imaging-based diagnosis in congenital coronary anomalies.

## Case report

A 3-year-old male patient, with no relevant prenatal, perinatal, or family history, presented with episodes of paroxysmal tachycardia since the age of 2 years and 6 months, New York Heart Association functional class III dyspnoea (Ross classification), and poor weight gain. He was referred for cardiological evaluation, and initial transthoracic echocardiography revealed marked left ventricular dilatation and severe functional mitral regurgitation. Treatment with spironolactone, furosemide, and captopril was initiated. Owing to persistence of symptoms, he was referred to a national tertiary referral centre for further evaluation and specialised management.

**Figure 1:**
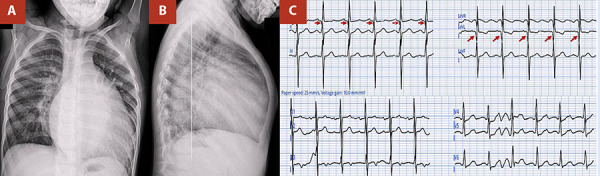
(A,B) Anteroposterior and lateral chest radiographs show cardiomegaly (cardiothoracic ratio >0.6) with pulmonary overcirculation. (C) The electrocardiogram demonstrates pathological Q waves in leads I and aVL, suggestive of a previous lateral myocardial infarction.

Physical examination showed mild tachypnoea, splitting of the second heart sound, and a grade III/VI holosystolic mitral murmur. Chest radiography demonstrated predominantly left-sided cardiomegaly and signs of pulmonary congestion. Electrocardiography revealed left ventricular hypertrophy and pathological Q waves in leads I and aVL (Figure 1). Transthoracic echocardiography confirmed severe mitral regurgitation secondary to ventricular dilatation, a preserved left ventricular ejection fraction (LVEF) of 58%, and an estimated pulmonary artery systolic pressure (PASP) of 70 mmHg, consistent with a high probability of pulmonary hypertension, with no other associated cardiac anomalies (Table 1).

**Table 1:** Evolution of structural and functional parameters assessed by echocardiography and cardiac computed tomography before and after surgery.

Parameter	Preoperative transthoracic echocardiography	Preoperative cardiac CT angiography	Immediate postoperative transthoracic echocardiography	Transthoracic echocardiography (postoperative day 7)	Transthoracic echocardiography (first postoperative year)	Transthoracic echocardiography (second postoperative year)
LV end-diastolic Z score	+5.7	+4.5	+3.8	+3.6	+2.3	+1.3
LV end-systolic Z score	+5.3	+4.1	+3.2	+3.7	+2.0	+1.2
LVEF (%)	58	-	32	50	66	70
RCA Z score	+2.1	+0.5	+0.6	+0.5	+1.36	+1.76
LCA trunk Z score	+3.8	+3.9	+3.8	+3.8	+2.5	+3.5
Probability of pulmonary hypertension (PASP, mmHg)	High (70)	-	Low (40)	Low (35)	Low (32)	Low (20)
Mitral regurgitation	Severe	-	Mild	Mild	Mild	Mild
Indexed left atrial area (cm²/m²)	70	85	51.3	14.7	12.3	11.4
Mitral annulus Z score	+4.8	+4.8	+2.0	+1.9	+1.0	+0.3

LV: left ventricle. LVEF: left ventricular ejection fraction. RCA: right coronary artery. LCA trunk: left coronary artery trunk. PASP: pulmonary artery systolic pressure. CT: computed tomography

Z-score values between -2 and +2 are considered within the normal range; values >+2 indicate increased size, and values <-2 indicate reduced size.

**Figure 2:**
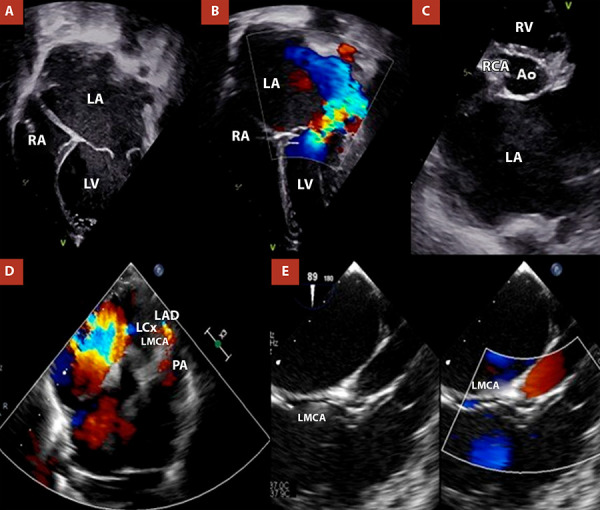
Transthoracic echocardiography **(A, B)** shows severe left atrial dilatation and severe mitral regurgitation. **(C, D)** The right coronary artery (RCA) is seen arising from the aorta (Z score +2.6), whereas the left main coronary artery (LMCA) originates from the pulmonary artery and bifurcates into the left circumflex artery (LCx) and the left anterior descending artery (LAD). Transoesophageal echocardiography **(E)**, bicaval view, demonstrates the anomalous course of the LCT arising from the pulmonary artery.

**Figure 3:**
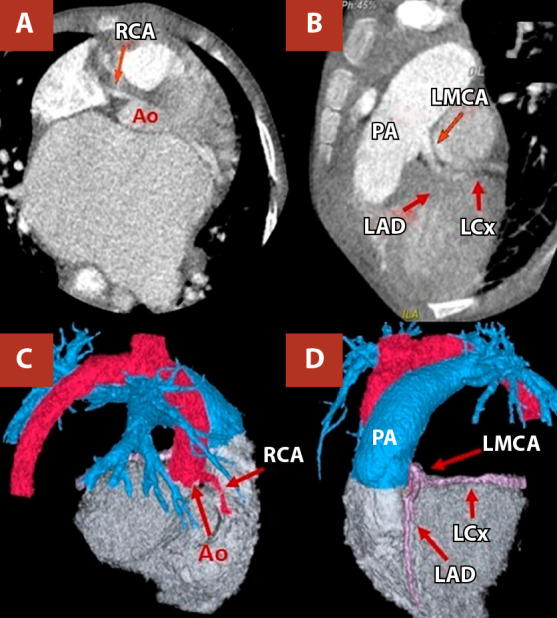
Coronary computed tomography angiography. **(A)** Normal origin of the right coronary artery from the ascending aorta. **(B)** The left main coronary artery arises anomalously from the pulmonary artery. **(C, D)** Three-dimensional reconstruction shows the normal course of the right coronary artery and the anomalous origin of the left main coronary artery

Given suspicion of a structural heart disease, transoesophageal echocardiography was performed, demonstrating an anomalous origin of the left coronary artery from the pulmonary artery (ALCAPA) (Figure 2). Cardiac computed tomography (CT) angiography confirmed the diagnosis and showed dilatation of the left atrium, mitral annulus, and main pulmonary artery with its branches (Figure 3, Table 1).

LA. left atrium. RA: right atrium. LV: left ventricle

RCA: right coronary artery. PA: pulmonary artery. LMCA: left main coronary artery. LAD: left anterior descendent. LCx: circunflex artery

The patient underwent surgical correction under cardiopulmonary bypass (approximately 4 h 50 min) and aortic cross-clamping (2 h 20 min), with Custodiol cardioplegia. The pulmonary artery was opened, identifying the anomalous origin of the left coronary trunk from the posterior aspect of the pulmonary sinus of Valsalva. The coronary artery was carefully dissected, fully mobilised, and reimplanted into the left aortic sinus of Valsalva, using an autologous pericardial patch to extend the coronary button (modified Cabrol technique). This was followed by posterior mitral annulus reduction annuloplasty using a continuous suture, without placement of a prosthetic ring or band, together with left atrial reduction and resection of the left atrial appendage. The procedure concluded with reconstruction of the pulmonary trunk and uneventful aortic declamping. Intraoperative transoesophageal echocardiography demonstrated mild residual mitral regurgitation, septal and left ventricular free wall hypokinesia, and an LVEF of 32% under dobutamine support at 5 μg/kg/min.

Postoperatively, the patient was extubated within the first 24 hours, with gradual weaning of inotropic support by postoperative day 4. Echocardiography on day 3 showed an LVEF of 43% with minimal mitral regurgitation. By day 7, LVEF had improved to 50%, accompanied by a progressive decline in troponin T (peak 2.3 ng/mL) and CK-MB (peak 88.6 U/L) to normal values, with no ischaemic changes on electrocardiography.

**Figure 4:**
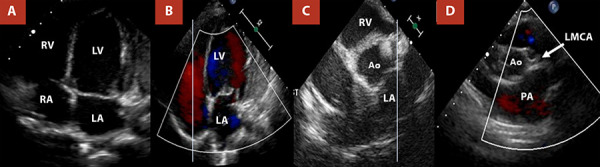
Follow-up transthoracic echocardiography at 2 years. **(A)** Reduction in left atrial size and decreased left ventricular diameter. **(B)** Minimal residual mitral regurgitation. **(C, D)** The left coronary trunk has been reimplanted into the left coronary sinus, with no diastolic reverse flow into the pulmonary artery.

He was discharged on postoperative day 8, in functional class I, with favourable clinical evolution. Treatment with captopril 6.25 mg every 12 hours and furosemide 10 mg daily was continued for 15 months. Owing to the COVID-19 pandemic, follow-up at 3 and 6 months was not performed; however, evaluations at 1 and 2 years were completed. At the latter visit, the patient was asymptomatic, with appropriate somatic growth. Echocardiography showed mild mitral regurgitation, LVEF of 70%, slightly reduced global longitudinal strain (−15%), anteroseptal hypokinesia, and reverse remodelling of the left ventricle (Figure 4, Table 1). Electrocardiography demonstrated negative T waves in leads V1-V4, without pathological Q waves.

RV: right ventricle. LV: left ventricle. RA: rigth atrium- LA: left atrium. LMCA: left main coronary artery. PA: pulmonary artery

## Discussion

ALCAPA is usually diagnosed in infancy due to symptoms of heart failure, although some cases are identified in adulthood, facilitated by partial collateral circulation ^5^. Nevertheless, the risk of ischaemia, arrhythmias, and sudden cardiac death persists even in oligosymptomatic patients ^6^. Our patient exhibited compatible manifestations, although the initial diagnosis was left ventricular dilatation and severe secondary mitral regurgitation of unclear aetiology.

Physical examination revealed a mitral systolic murmur and signs of pulmonary congestion, reflecting left ventricular dysfunction and secondary mitral regurgitation, characteristic features of this condition ^1^^,^^3^. The initial electrocardiogram showed left ventricular hypertrophy and pathological Q waves in leads I and aVL, indicative of chronic anterolateral ischaemia ^7^. Chest radiography demonstrated cardiomegaly and pulmonary overcirculation, findings frequently described in paediatric ALCAPA series ^3^.

Transthoracic echocardiography is the first-line diagnostic modality for ALCAPA, with a reported accuracy of 90.9% in paediatric series ^8^. However, sensitivity may decrease in complex anatomies or in the presence of poor acoustic windows ^8^. Echocardiographic findings include direct signs, such as visualisation of the anomalous origin of the left coronary artery and retrograde flow into the pulmonary artery, and indirect signs, including left ventricular dilatation, functional mitral regurgitation, right coronary artery dilatation, papillary muscle fibrosis, and a right coronary artery-to-aortic annulus ratio >0.14 ^9^. In our case, severe left ventricular dilatation and severe mitral regurgitation were initially identified; subsequent reassessment demonstrated retrograde flow in the left coronary artery, supporting the diagnosis of ALCAPA.

In neonates with ALCAPA, elevated pulmonary arterial pressure may temporarily maintain anterograde flow from the pulmonary artery to the left coronary artery, masking myocardial ischaemia ^3^^,^^5^. Our 3-year-old patient exhibited an advanced haemodynamic pattern, consistent with the natural history of untreated ALCAPA, characterised by retrograde flow from the left coronary artery to the pulmonary artery, collateralisation from the right coronary artery, and echocardiographic signs of pulmonary hypertension (estimated pulmonary artery systolic pressure of 70 mmHg).

Coronary CT angiography is the imaging modality of choice to accurately define coronary origin and course in congenital anomalies, having largely replaced diagnostic catheterisation ^10^. Cardiac magnetic resonance imaging allows assessment of ventricular function and tissue characterisation with late gadolinium enhancement; in paediatric ALCAPA, the presence of myocardial scar has been associated with slower recovery and a higher risk of reintervention, underscoring its prognostic value ^11^. In our patient, coronary CT angiography confirmed the origin of the left coronary artery from the posterior left pulmonary sinus. Cardiac magnetic resonance was not deemed necessary, as preoperative left ventricular ejection fraction was preserved and imaging results would not have altered the surgical indication. Moreover, the clinical course was favourable, with preserved ventricular function and absence of symptoms or tachyarrhythmias.

Surgical treatment of ALCAPA is universally indicated, given the high risk of myocardial ischaemia and sudden death, even in asymptomatic patients ^12^. The goal is to restore a dual coronary circulation, preferably through direct reimplantation of the left coronary artery into the aorta, which is the technique of choice when anatomy permits ^10^. In complex anatomies, alternative techniques may be required, including the Takeuchi repair, coronary artery bypass grafting, or the modified Cabrol technique ^13^. In our case, the latter was employed due to anatomical complexity that precluded tension-free direct reimplantation ^14^.

Mitral regurgitation, frequently observed in ALCAPA, is usually functional and secondary to chronic ischaemia of the subvalvular apparatus ^15^, with papillary muscle fibrosis demonstrated on magnetic resonance imaging ^16^. Although mitral regurgitation may improve after revascularisation, concomitant surgical correction is recommended in cases with significant regurgitation ^17^. In this patient, mitral annuloplasty was performed during the same surgical procedure, resulting in favourable clinical and echocardiographic outcomes.

Operative mortality ranges from 0% to 16% ^18^, and long-term survival may reach 86% ^19^. However, the risk of late sudden cardiac death due to residual fibrosis and ventricular arrhythmias persists, highlighting the need for structured long-term follow-up ^20^. In our case, postoperative evolution was favourable, with progressive recovery of systolic function, mild residual mitral regurgitation, and functional class I at follow-up.

The main limitation was the absence of early postoperative echocardiographic follow-up, which limited assessment of initial recovery. Diastolic function was also not evaluated, given the lack of standardised parameters in paediatric patients with congenital heart disease.

In conclusion, this case underscores the importance of maintaining a high index of suspicion for ALCAPA in paediatric patients presenting with left ventricular dilatation and severe mitral regurgitation of unclear aetiology, as well as the significant impact of timely surgical intervention and structured follow-up on prognosis.
